# Sensing Method Using Multiple Quantities for Diagnostic of Insulators in Different Ambient Conditions

**DOI:** 10.3390/s22041376

**Published:** 2022-02-11

**Authors:** Bystrík Dolník, Ľuboš Šárpataky, Iraida Kolcunová, Peter Havran

**Affiliations:** Department of Electric Power Engineering, Faculty of Electrical Engineering and Informatics, Technical University of Košice, Letná 9, 04200 Košice, Slovakia; lubos.sarpataky@tuke.sk (Ľ.Š.); iraida.kolcunova@tuke.sk (I.K.); peter.havran@tuke.sk (P.H.)

**Keywords:** insulator, pollution, dielectric loss factor, leakage current, capacity, electric charge, humidity

## Abstract

Insulators are one of the many components responsible for the reliability of electricity supply as part of transmission and distribution lines. Failure of the insulator can cause considerable economic problems that are much greater than the insulator cost. When the failure occurs on the transmission line, a large area can be without electricity supply or other transmission lines will be overloaded. Because of the consequences of the insulator’s failure, diagnostics of the insulator plays a significant role in the reliability of the power supply. Basic diagnostic methods require experienced personnel, and inspection requires moving in the field. New diagnostic methods require online measurement if it is possible. Diagnostic by measuring the leakage current flowing on the surface of the insulator is well known. However, many other quantities can be used as a good tool for diagnostics of insulators. We present in this article results obtained on the investigated porcelain insulators that are one of the most used insulation materials for housing the insulator’s core. Leakage current, dielectric loss factor, capacity, and electric charge are used as diagnostic quantities to investigate porcelain insulators in different pollution conditions and different ambient relative humidity. Pollution and humidity are the main factors that decrease the insulator´s electric strength and reliability.

## 1. Introduction

Insulators are pieces of electrical equipment to support electrical conductors on transmission and distribution towers and to separate them electrically from equipment that must not be under voltage. Insulators are divided according to the used insulation material, insulator´s construction, or used fittings. The most used insulation materials are glass, porcelain, and composite materials. The most used composite material is silicon rubber. All these materials have their advantages and disadvantages. The choice of a suitable insulator depends on many factors. The correct insulator to be used for the specific application is determined by the operating voltage, the mechanical load, and environmental factors such as pollution, high humidity, wind, lightning strokes, and many others. Therefore, it is impossible to select one insulator design or material that will be most suitable for all applications and the environment [1,2,3,4,5,6].

Capacitive sensors have a wide range of usability. Capacitive sensors have been investigated to detect the aging of the silicone insulator. Experiments demonstrated that the proposed capacitive sensors exhibited excellent performance in signal amplitude, sensitivity, and stability against the effects of environmental humidity [7]. Other research concentrates on a flexible capacitive tactile sensor. Sensors were used to measure normal force, shear force, and torsion. Research proved that concentric-shape electrodes were enabled to avoid unaffectedly the normal force when measuring the pure shear force due to concentric-shape electrodes [8].

Diagnostic methods used for decades are based on a measurement that requires contact with the insulator by skilled personnel. A visual check is the most basic diagnostic method. This method is still one of the most reliable inspection techniques. However, it is almost impossible to find punctures that are not visible. Because of the disadvantages, the other techniques were tested. Measurement of electric field and voltage distribution on the insulators string, acoustic emission measurement, night vision camera, infrared thermography is used to improve the diagnostic of the insulator and reach a representative result. However, all of these techniques require qualified personnel who must be in contact with or near the insulator [9,10,11,12,13,14,15,16].

Researchers nowadays started to use information technology to analyze the problematics of insulators. Fault, puncture, or contamination of the insulator surface can be analyzed, simulated, or predicted using various software or software procedures. Recent studies with numerical simulation models of insulators show that numerical techniques are valid for calculating the electric field and potential distribution on insulators, such as the boundary element method (BEM), the finite difference method (FDM), and the finite element method (FEM) [17,18,19].

Another piece of research focused on the classification of insulators using neural networks based on computer vision. The result shows that the contaminated insulators classification can reach 97.5 % accuracy if the neural network model is used [20].

Other researchers work with ultrasound equipment that can help to forecast insulator contamination. With the correct measurement and information technology set, it is possible to predict contamination with good accuracy of a signal with many nonlinearities [21,22].

When the insulator is under high voltage, leakage current passes through the insulator’s surface. The magnitude of the leakage current depends on surface conditions and ambient environmental conditions. In case the surface of the insulator is damaged by the aging process, the leakage current flowing through the surface of the insulator increases. Furthermore, the increase in leakage current accelerates the aging process. The next factor that influences the leakage current is contamination. The contamination is dangerous for insulators, especially when it is wetted. In a wet and polluted environment, the leakage current increases rapidly and causes damage to the insulator’s surface. Long-term leakage current activity causes punctures, tracking, or erosion on the insulator’s surface. Long-term leakage current measurement can indicate an aging process of the insulator and a reduction in its dielectric strength [2,23,24,25].

Leakage current measurement is well known and often used as an inspection technique. Measurement leakage current is one of the non-destructive diagnostic methods if we compare it with methods that require voltage higher than the rated voltage. The leakage current measurement can indicate the problems on insulators. It is used predominantly for contamination inspection. However, the leakage current measurement is more useful for applications with only one insulator. However, there are successful studies that measure leakage current on insulators string. N.A. Othman et al. use a shunt resistor connected to the grounded end to measure leakage current on glass insulator string and results show significant changes with increasing contamination [23]. Another successful measurement was done on non-coated, half-coated, and full-coated insulator strings [26]. The pollution layer on the surface of the insulator in a humid environment in any form increases the leakage current that flows on the surface of the insulator. The probability of flashover can be indicated by measuring the leakage current if sufficient data is available. From the practical point of view, the leakage current measurement does not need special equipment and can be measured online. The amplitude of the leakage current increases according to the relative humidity increase. However, the combination of contamination and humidity largen leakage current value even more [27,28,29,30,31,32].

The detailed measurement that uses Fourier transformation to investigate harmonic compound shows that third, fifth, and seventh harmonic compound of leakage current increases with pollution level. The main harmonic compound is mostly affected by humidity. Researches focused on the leakage current harmonic compounds shows the statistical treatment that indicates the probability of failure and the necessity of the insulator’s maintenance. Researchers use harmonic indexes to evaluate the possibility of flashover. The difference between indexes is a ratio between harmonic compounds [27,32,33,34,35,36,37].

Only a few studies have used a loss factor to indicate the aging of insulators. The loss factor measurement is mainly used to diagnose transformers, cables, and windings. Results show the loss factor values correlate with aging. So, the loss factor can indicate the degradation of insulating material. Another finding confirms that small changes in applied voltage do not affect the dielectric loss factor value [38,39].

After studying the problematics of diagnostics of insulators, the main goal nowadays is to indicate pollution level and probability of flashover as accurately as possible. Regular flashover testing is not appropriate because of the degradation of insulating materials and accelerated aging. The measurement of quantities measured at low voltage is safer and can be investigated at any time or online. Many types of research are based on the measurement of leakage current. Leakage current measurement is a suitable diagnostics method to investigate the impact of pollution and humidity.

However, few types of research are focused on other quantities that do not need high voltage for measurement. In this paper, the dielectric loss factor, capacity, and electric charge are used to investigate the influence of pollution and humidity on glazed porcelain insulators. According to the search of a publication focused on diagnostics of insulators, no research uses these quantities to diagnose the influence of pollution and humidity on insulators. These quantities can measure at low voltages as leakage current. Results of these quantities are compared to leakage current measurement to indicate the suitability of the use of quantities.

Moreover, the multiple quantities measurement could be a good indicator for the risk of pollution flashover because of more verifications of results. This measurement uses frequencies that are higher than the standard frequency. It helps to avoid the problem with electromagnetic interference that becomes negligible. This helps to improve the online monitoring reliability of used quantities. Some quantities are more useable in the low frequencies, some of them show better results in high frequencies.

Sensing electrodes are prepared by technology that has not been used for the investigation of pollution on insulators, and they are suitable for practical use in any ambient conditions. Research is focused on the changes in quantities according to surface conditions and ambient relative humidity conditions. A clean insulator simulates the beginning of the life cycle of the insulator and pollution levels simulate consecutive contamination of the insulator through its lifespan. Measurement of multiple quantities for pollution investigation could show more significant differences between pollution levels and humidity rate and should be more suitable for pollution investigation.

## 2. Materials and Methods

**Figure 1:**
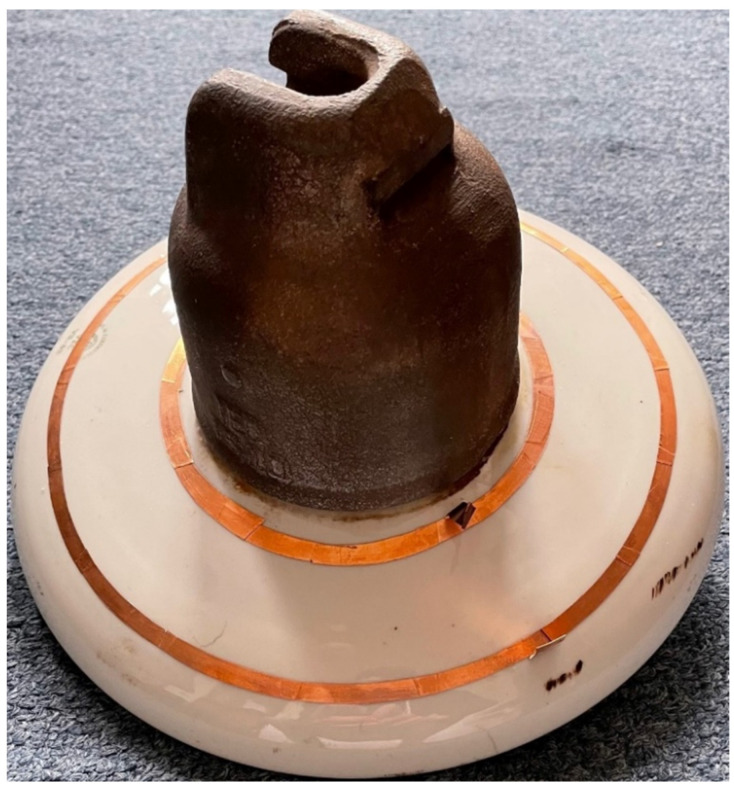
Porcelain insulator with sensing electrodes.

The laboratory is located at the Technical University of Košice, where all measurements were performed. The porcelain insulator has two circular sensing electrodes. The gap between sensing electrodes made from conducting tape is 4 cm. The diameter of the inner electrode is 15 cm, and the diameter of the outer electrode is 23 cm. The total area between the electrodes is 238 cm^2^. In Figure 1, the insulator with sensing electrodes is depicted.

**Figure 2:**
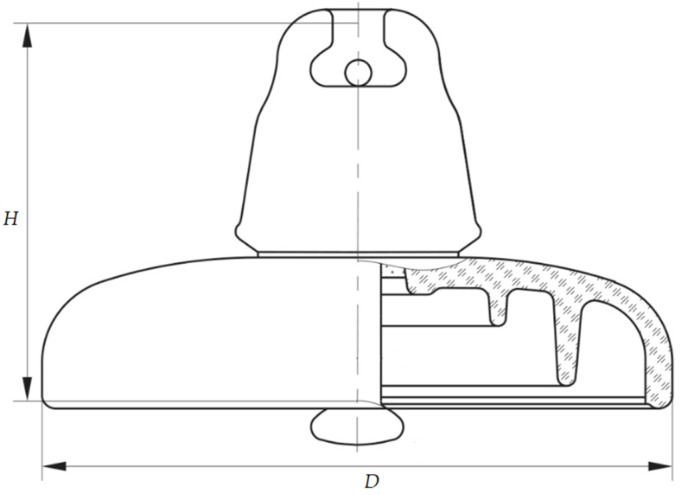
Size parameter of porcelain insulator U 160 BL.

The porcelain insulator shown in Figure 1 is suitable for high-voltage transmission lines as a part of the insulator’s string. The shed of the insulator is glazed. The porcelain insulator was made in the factory Elektroporcelán Louny in the Czech Republic. According to IEC standard 60305:2021, the insulator is U 160 BL type. The diameter (*D*) is 280 mm, and spacing (*H*) is 170 mm. In Figure 2, the size parameter of the insulator is depicted. The minimum creepage distance of the insulator is 340 mm, and the mechanical failing load is 160 kN.

**Figure 3:**
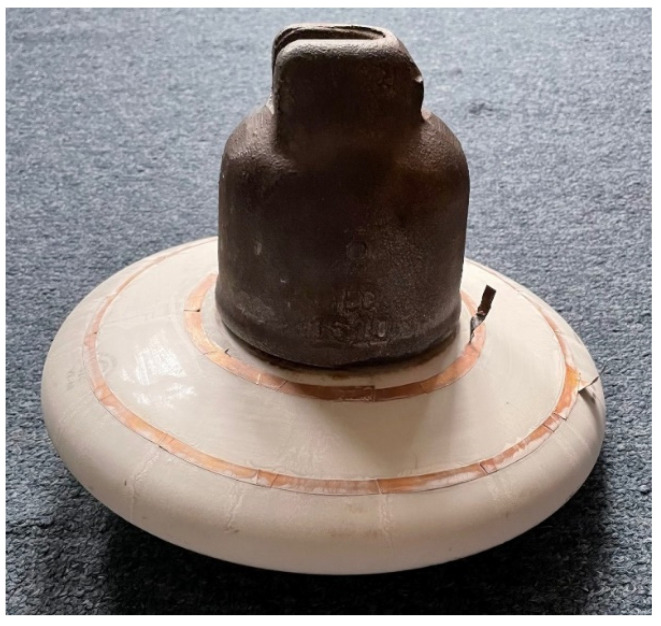
Porcelain insulator contaminated by second pollution level (L2).

The sensing electrodes were used to measure leakage current, dielectric loss factor, capacity, and electric charge. All quantities were measured at an ambient relative humidity of 40 to 90%. Four pollution levels were prepared to simulate different pollution severities according to IEC standard IEC/TS 60815-1:2008. We marked the prepared pollution levels with abbreviations (L1, L2, L3, L4). The individual solutions were prepared by mixing a specified amount of salt, kaolin, and tap water. The kaolin in tap water creates an even distribution of the contaminating layer on the surface of the insulator. For each level of contamination, an equal amount of kaolin was added to the solution. The amount of kaolin is 40 g for each liter of water. The individual pollution levels with the amount of salt added per liter of tap water are in Table 1. In Figure 3, the porcelain insulator contaminated by the second pollution level is depicted.

Measurements for every pollution level started at a relative humidity of 40% in a closed chamber. If relative humidity at the beginning was lower than 40%, the increase of relative humidity started. After the relative humidity reached the required value, a waiting period of 10 min started to ensure the surface of the porcelain insulator absorbs the humidity. After 10 min, the absorption of humidity by pollution level slows down to the minimum and it has a minimum influence on the measured values. After a measurement that lasted approximately 7 min, the relative humidity gradually increased to the next desired value. After reaching the next required level, the procedure was repeated. 

**Figure 4:**
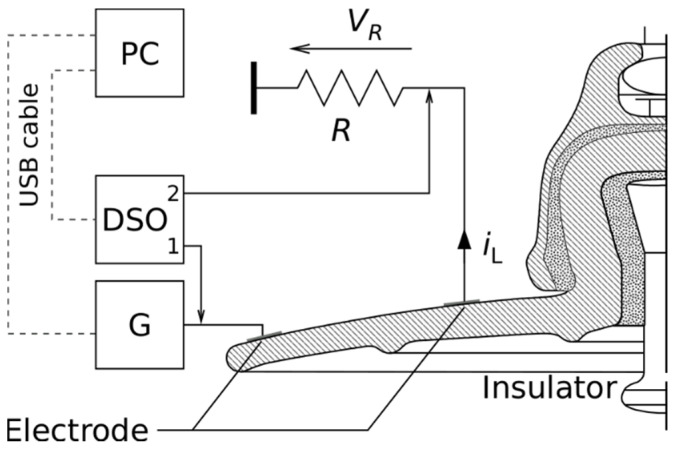
Leakage current and electric charge measurement diagram.

Leakage current and electric charge measurement and calculations applied on porcelain insulators in various pollution conditions and relative humidity from 40 to 90% were done in the insulated chamber. On sensing electrodes, function/arbitrary waveform generator Agilent 33210A (G) and an oscilloscope Agilent DSO7104A (DSO) were connected. We used two different frequencies of the testing voltage applied on sensing electrodes. The first frequency of the testing voltage, close to the power frequency, is 113 Hz. The second frequency, several times greater than the power frequency, is 1 kHz. These two frequencies, different from power frequency, prevent electromagnetic interference from the supply network. The resulting leakage current (*i*_L_) r.m.s value was calculated according to Ohm’s law as the average of 128 periods of voltage drop (*V_R_*) on the non-inductive sensing resistor (*R*) divided by the resistance value. A rectangular testing voltage was applied to the measuring electrodes to evaluate the electric charge transferred through the surface of the porcelain insulator. The electric charge was calculated numerically using the rectangle rule. A personal computer (PC) connected to the measuring devices via a USB port controlled the frequency of the test voltage, recorded the time course of the leakage current, and stored the measured data. The script was written in the Python programming language in the Linux operating system under Ubuntu distribution used to collect the measured data from the oscilloscope and calculate the electric charge. The measurement diagram of leakage current and electric charge is in Figure 4. The numbers 1 and 2 in the DSO diagram indicate the channels used.

Capacity (*C*) and dielectric loss factor (tan *δ*) were measured with an impedance analyzer HIOKI LCR meter IM3533-1(Fotronic Corporation, Woburn, MA, United State). The impedance analyzer can measure different quantities in a wide range of frequencies from 1 mHz to 200 kHz. We used a connection with four individual shielded coaxial cables with a length of 1 m. Before every measurement series, calibration of the measurement device was performed. To achieve the accuracy declared by the LCR meter manufacturer, open and short circuit calibration was performed on the connected coaxial cables. Calibration was performed according to the manufacturer’s recommendations. After successful calibration, the test cables were connected to the electrodes on the surface of the porcelain insulator in a closed chamber.

**Figure 5:**
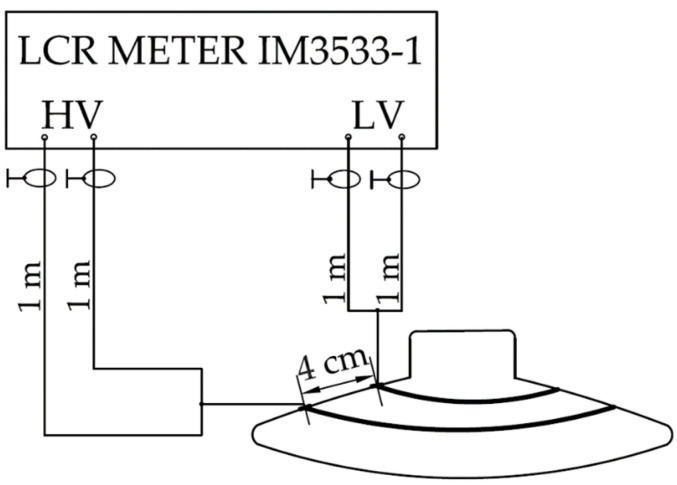
Capacity and dielectric loss factor measurement diagram.

The LCR meter measured capacity and tan *δ* simultaneously with a measuring voltage of 5 V swept from 1 Hz to 200 kHz. The frequency range was divided into 100 points on a logarithmic scale, with 20 measurements averaged at each frequency point. The circuit diagram of the impedance analyzer connected to the measuring electrodes using coaxial cables is shown in Figure 5.

Dielectric loss factor is a tangent of the dielectric loss angle (*δ*) between two vertical components, namely, the capacity current *I*_C_ and the resistance current *I*_R_. The third current component (*I*_p_) influencing the loss angle represents polarization of dielectric.

(1) $$\tan\;\delta =\frac{I_{R}}{I_{C}}=\frac{\frac{U}{R}}{\omega \cdot C\cdot U}=\frac{1}{\omega \cdot C\cdot R}$$

The dielectric loss factor is influenced by the conductivity of the insulation. As none of the insulation is perfect, not only a reactive component of current is present but an active component is present as well due to conductivity and polarization of dielectric. Humidity and pollution increase the conductivity of the surface of the insulator [40].

Polarization processes occur in a material because of the restricted movement of charges. Nevertheless, charges are bound, they can be displaced from their equilibrium position. An electric field that is applied cause two types of polarization. The first is rapidly forming polarization, which does not cause any dielectric losses. The second is slowly forming polarization, also called relaxation type of polarization. Interfacial, or migration, polarization (Maxwell-Wagner effect) takes place in heterogeneous materials, containing conducting components. The relaxation time of interfacial polarization is *τ* = 10^−6^–10^−3^ s. Another type of relaxing type of polarization is induced polarization or electrochemical polarization. It occurs in the electrical double layers on the boundaries between the solid and liquid phases. This interface causes a potential difference to develop. Thus, the interface becomes electrified [41]. The polarization can affect the dielectric loss factor especially when the surface is polluted and wetted.

## 3. Results

The measurement of all quantities was performed first on a clean porcelain insulator, then on four different pollution levels of the insulator surface. Because of two measurement schemes, the insulator was measured in two steps. The first measurement was performed according to the scheme shown in Figure 4. The increase in relative humidity vitiates the pollution layer on the surface of the insulator. Because of this fact, after the measurement of leakage current and electric charge, the insulator was precisely cleaned. After cleaning, the pollution layer was recreated to measure the dielectric loss factor and capacity according to the scheme shown in Figure 5.

### 3.1. Measurement of Dielectric Loss Ffactor in the Frequency Range from 1 Hz to 200 kHz

**Figure 6:**
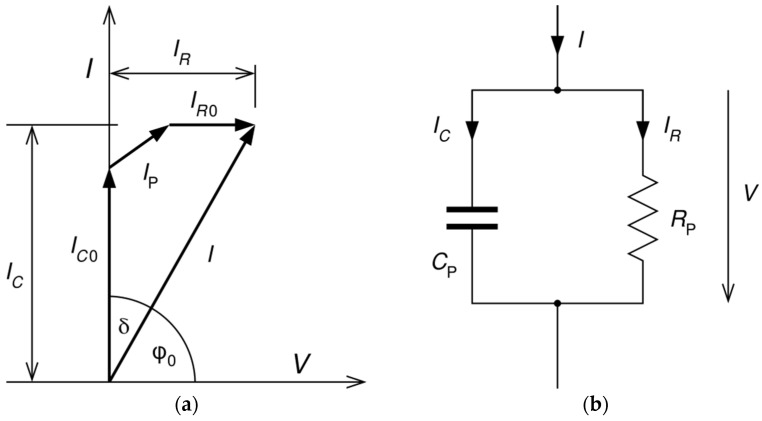
Dielectric loss factor: (**a**) vector diagram; (**b**) simplified equivalent circuit.

We present the results from the measurement of dielectric loss factor under different pollution levels and on the clean insulator in Figure 6.

**Figure 7:**
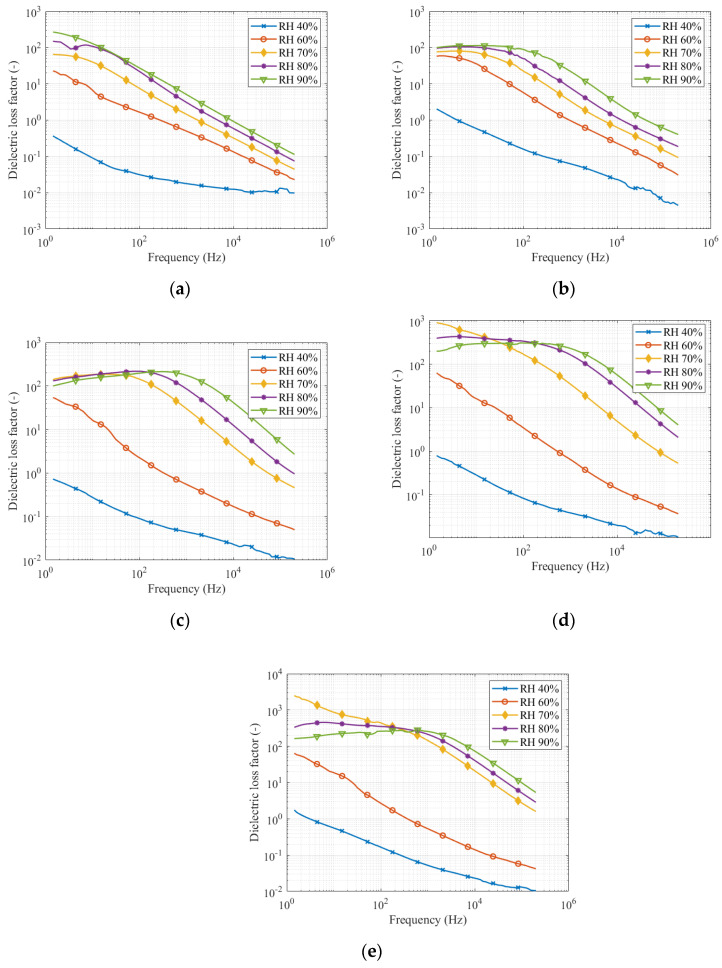
Dielectric loss factor in the frequency range: (**a**) clean insulator; (**b**) first pollution level; (**c**) second pollution level; (**d**) third pollution level; (**e**) fourth pollution level.

Measurement results in Figure 7 show that the dielectric loss factor increases with increasing relative humidity. It is clear from Figure 7 that with the increase in pollution levels, the dielectric loss factor increases. The frequency response of the dielectric loss factor in Figure 7a,b under a relative humidity of 40% is lower than under higher values of relative humidity. Measurements with a relative humidity of 60% and higher are more “grouped” and the difference between them is not that significant. For the second, third, and fourth pollution levels, in Figure 7c–e, the rapid increase of dielectric loss factor is visible between 60% of relative humidity and higher relative humidity. So, the highest dielectric loss factor increase is between 60 and 70%.

The increase in dielectric loss factor between these two relative humidities is at a frequency of 1 kHz, from 51 times to 263 times higher for different pollution levels. For the fourth pollution level, the dielectric loss factor for 60% relative humidity is 0.48, and for relative humidity, 70% is 126.5.

The other important information seen in Figure 7 shows that the local maximums or the peak values are moving to the higher frequencies with increasing pollution levels. For measurement at 90% relative humidity, dielectric loss factor peak value changes from 276 at frequency 1 Hz for clean insulator, to 214 at frequency 256 Hz for the second pollution level, finally to 307 at frequency 475 Hz for the fourth pollution level. The peak value of the dielectric loss factor is changing with relative humidity too. For the second level of pollution, frequency changes from 1 Hz for 40 and 60% relative humidity, to 15 Hz for 70% relative humidity, to 74.8 Hz for 80% relative humidity, and finally to 256 Hz for 90% relative humidity. 

### 3.2. Measurement of Capacity in the Frequency Range from 1 Hz to 200 kHz

**Figure 8:**
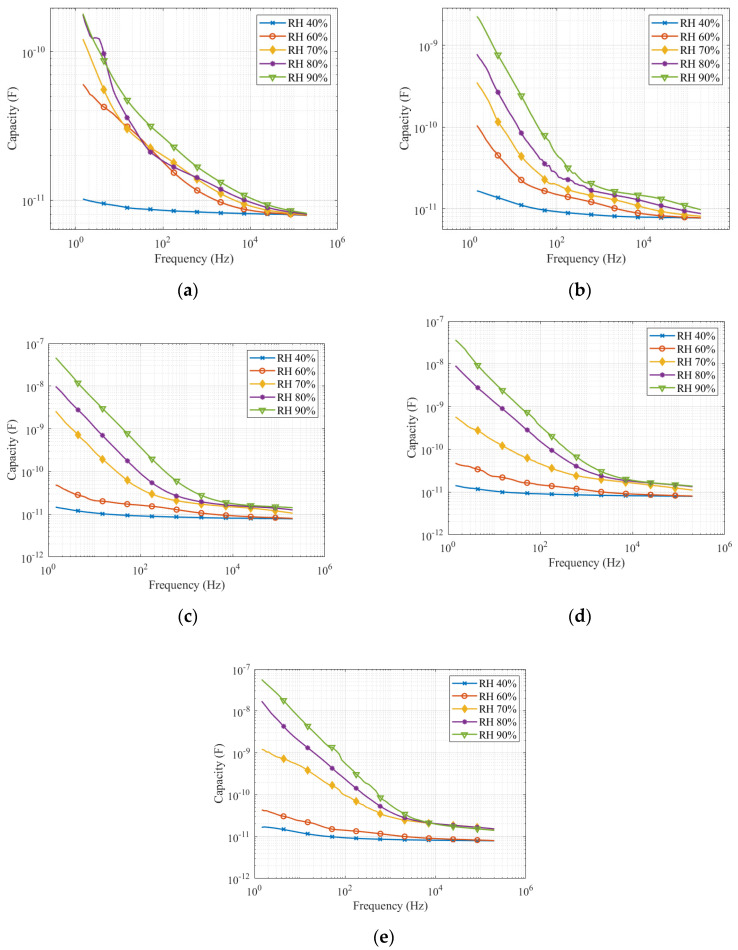
Capacity in the frequency range: (**a**) clear insulator; (**b**) first pollution level; (**c**) second pollution level; (**d**) third pollution level; (**e**) fourth pollution level.

In Figure 8, we present the results of measured capacity under different pollution levels and on the clean insulator.

Measurement results of capacity in a wide frequency range show the capacity decrease with increasing frequency. Figure 8 shows a significant capacity dependence on relative humidity for all pollution levels. The difference between the individual measured capacities for different relative humidities is evident in the low-frequency range. For frequencies higher than 1000 Hz, the curves have converged and the difference is not that obvious. As seen in Figure 8, the measured capacity at 40% relative humidity is significantly lower than the capacity at higher relative humidity for clean insulators and the first level of contamination. In the case of higher pollution levels, capacity frequency response at a relative humidity of 40 and 60% are grouped. The measured capacity at the relative humidity of 70, 80, and 90% for the second, third, and fourth pollution levels is significantly higher and almost evenly distributed.

### 3.3. Comparison of All Quantities at the Frequency 113 Hz and 1 kHz

The leakage current measurement and electric charge numerical evaluation were performed for frequencies 113 and 1000 Hz. The dielectric loss factor and capacitance are taken from the frequency response measurements in Figure 7 and Figure 8. The measured data are in Table 2, Table 3, Table 4 and Table 5.

**Figure 9:**
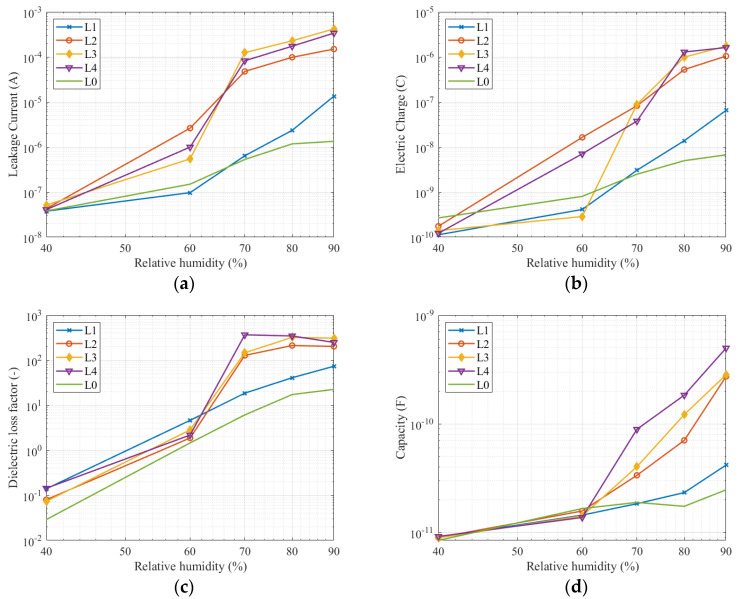
Measured quantities at frequency 113 Hz: (**a**) leakage current; (**b**) electric charge; (**c**) dielectric loss factor; (**d**) capacity.

The dependencies in Figure 9 and data in Table 2, Table 3, Table 4 and Table 5 show measurement results at frequency 113 Hz of the testing voltage. All measured quantities show a rapid increase of measured values for the second, third, and fourth pollution levels after an increase in relative humidity from 60 to 70%. Leakage current and dielectric loss factor increase between 70 and 80% relative humidity are slight and constantly increasing to 90% relative humidity. The capacity increase is slight up to 60% relative humidity, then increases steeply. The measured electrical quantities on a clean insulator and with the first level of pollution increase more and more, except the electric charge as for each pollution level, the electric charge rises sharply between 60 and 80% relative humidity. Leakage current, electric charge, and capacity show a significant difference between the clean insulator and first level of pollution and other pollution levels at relative humidity 70% and higher. Dielectric loss factor is only one quantity where clean insulator has lower values than for first pollution level for a relative humidity 40 and 60%.

**Figure 10:**
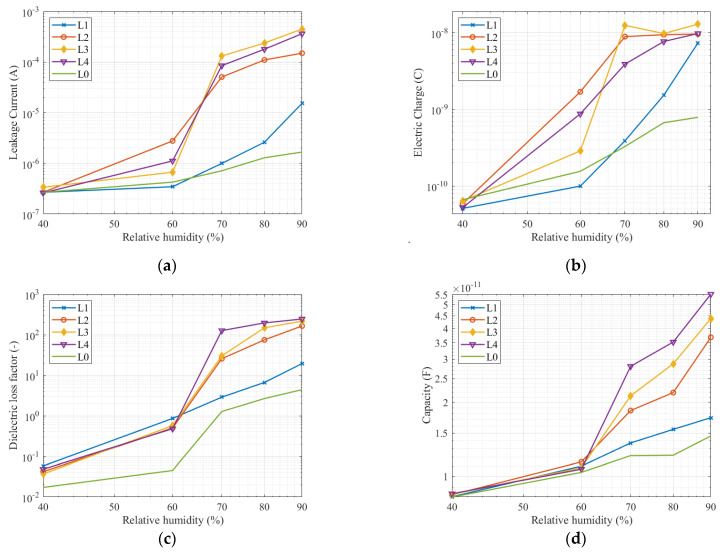
Measured quantities at frequency 1 kHz: (**a**) Leakage current; (**b**) Electric charge; (**c**) Dielectric loss factor; (**d**) Capacity.

The dependencies in Figure 10 and data in Table 2, Table 3, Table 4 and Table 5 show measurement results at frequency 1 kHz of the testing voltage. Measured quantities show the same trend as measurement at frequency 113 Hz. Measured quantities increase with relative humidity increase. The measured quantities on a clean insulator and the first pollution level are close together. Similarly, the second grouping consists of measured quantities for the second, third, and fourth pollution levels. Capacity measurement shows the most relevant differences between pollution levels. Capacity values increase gradually and evenly. The shape of curves of all quantities seems to have the same progress as the curves measured at frequency 113 Hz.

## 4. Discussion and Conclusions

Perspective multiple quantities diagnostic method, to indicate the pollution level on the surface of insulators, has been introduced and described in this work. All quantities point out the change in values with increasing relative humidity and pollution level. Sensing electrodes on the porcelain insulator were polluted several times without failure during the measurement was performed. Averaging multiple measured quantities were set in the measurement procedure to avoid erroneous data.

Measurement results show that all the quantities are acceptable as an indicator of pollution level. From Figure 9 and Figure 10, it is evident that the most relevant results were between relative humidity 70 and 80%. This fact can be checked in Table 2, Table 3, Table 4 and Table 5. Due to the wide range of measured values in the figures, the difference between the higher pollution level is not significant. However, the difference between light pollution (the first pollution level) and other pollution levels, which belong to medium and heavy pollutions are evident and can be identified. All quantities rapidly increase between relative humidity 60 and 70%. The increase in leakage current, electric charge, and dielectric loss factor slow down between 70 and 90% relative humidity. Only the capacity increases sharply up to a relative humidity of 90%. 

If we compare a clean insulator with contaminated insulators, the increase of measured quantities depending on the relative humidity is not so significant for an insulator with a clean surface. This observation is due to the ability of the solution to absorb water from the air. Experiments published in [42] show that the saturation on the contaminated layer depends on the contaminant layer composition on the surface of the insulator. As seen from the graphs in Figure 8 and Figure 9, there is a significant saturation between 60 and 70% relative humidity. In the case of using a solution with a different composition, a sharp increase in the measured quantities at a different relative humidity can be expected. Further studies dealing with pollution in various areas [43] have shown that the polluting layer may consist of different compounds having different physical properties (conductivity, hydrophilicity). Therefore, we recommend monitoring the measured values even at lower relative humidity.

The difference between the quantities measured at the second, third, and fourth pollution levels at a relative humidity of 70–90% are not as significant as between these pollution levels and the first pollution level. We have found that the higher the pollution level, the higher the effect of moisture on the pollution layer. Capacitance, leakage current, and electric charge are suitable for diagnostic measurements at one selected frequency. 

Because dielectric loss factor peaks are dependent on frequency when relative humidity and pollution are changed, it is appropriate to measure the dielectric loss factor in the frequency range to indicate the movement of the peak value. On the other hand, dielectric loss factor data for two chosen frequencies show the same trend as the other measured quantities. It could be helpful to select a frequency higher than 1000 Hz. The curves in Figure 6 seem to be more stable for frequencies higher than 1000 Hz. Measurement of quantities on clear insulators indicates lower resistivity than polluted insulators at low relative humidity. Saturation plays a key role in the presence of a polluted layer. The insulator becomes conductive after the pollution layer gets humid.

Leakage current measurements confirm results from the studied literature. Other measured quantities have the same trend as the leakage current trend, so measuring multiple quantities can help determine the severity of insulation pollution by specifying the pollution level more precisely.

Monitoring of the relative humidity seems to be very helpful for increasing reliability. According to IEC 60071: 2010, the reference value of relative humidity is 60% (11 g/m^3^). The significant change in the increase of the measured quantities is between 60 and 70% relative humidity, and then the increase is slight up to 90% relative humidity. Studied literature pointing out the most dangerous ambient condition for flashover is fog. Generally, the fog has higher relative humidity than 70% and the value is near 99%. According to our results, the danger of failure starts at a relative humidity of 70% reached in temperate weather conditions. The measurement of pollution levels under various relative humidity shows the necessity of monitoring the insulators not only in extreme conditions.

The advantage of the porcelain insulator with measuring electrodes used in the experiments was that measuring the leakage current of the porcelain insulator is not necessarily required to suspend in a power frequency supply network. The power supply network contains a certain percentage of higher harmonic components, which cause errors in evaluating the leakage current using frequency analysis. Another advantage is that the porcelain insulator with the measuring electrodes can be placed in any area to detect the degree of contamination. The diagnostic quantities measured by a suitable measuring system can be stored in a database and evaluated online. Subsequent analysis of the measured quantities indicates the possible need for an earlier cleaning period in critical areas of the power system and helps prevent insulator failure.

In the next step, we will continue examining other materials used for insulator sheds or housings like silicon rubber composite material. Moreover, samples of recycled composite materials are being prepared to compare them with standard materials. The experiments will be repeated on aged samples using accelerated aging to indicate the influence of the insulator aging on the measured quantities.

## Figures and Tables

**Table 1:** Information about salt amount and conductivity of four levels of artificial pollution.

Pollution Level	NaCl Amount (g/L)	Solution Conductivity (µS/cm)
L1	1	28
L2	6	129
L3	10	173
L4	16	286

**Table 2:** Leakage Current Measured Data at Frequencies 113 Hz and 1 kHz.

Leakage Current (μA)										
	Clean insulator		L1		L2		L3		L4	
RH (%)	113 Hz	1 kHz	113 Hz	1 kHz	113 Hz	1 kHz	113 Hz	1 kHz	113 Hz	1 kHz
40	0.004	0.368	0.004	0.228	0.004	0.268	0.005	0.338	0.004	0.261
60	0.150	0.426	0.01	0.346	2.62	2.77	0.546	0.670	0.998	1.12
70	0.530	0.714	0.64	0.996	47.7	50.7	124	131	82.2	85.0
80	1.18	1.29	2.35	2.61	98.2	109	228	238	173	179
90	1.34	1.66	13.4	15.3	149	149	417	448	338	362

**Table 3:** Electric charge evaluated data at frequencies 113 Hz and 1 kHz.

Electric Charge (nC)										
	Clean insulator		L1		L2		L3		L4	
RH (%)	113 Hz	1 kHz	113 Hz	1 kHz	113 Hz	1 kHz	113 Hz	1 kHz	113 Hz	1 kHz
40	0.367	0.066	0.113	0.051	0.175	0.058	0.139	0.064	0.122	0.052
60	0.804	0.155	0.411	0.096	16.4	1.69	0.286	0.287	7.06	0.872
70	2.49	0.328	3.05	0.389	82.3	8.87	88.4	12.4	37.6	3.86
80	4.99	0.668	13.8	1.524	89.4	9.4	992	9.73	128	7.66
90	6.71	0.785	65	7.329	107	9.6	1728	12.9	1615	9.74

**Table 4:** Dielectric loss factor measured data at frequencies 113 Hz and 1 kHz.

Dielectric Loss Factor (-)										
	Clean insulator		L1		L2		L3		L4	
RH (%)	113 Hz	1 kHz	113 Hz	1 kHz	113 Hz	1 kHz	113 Hz	1 kHz	113 Hz	1 kHz
40	0.029	0.017	0.14	0.058	0.08	0.042	0.073	0.037	0.14	0.048
60	1.46	0.045	4.63	0.86	1.88	0.5	2.84	0.572	2.18	0.48
70	6.1	1.28	18.6	2.88	128	25.6	146	30.1	367	126
80	17.3	2.66	40.9	6.64	212	74.6	322	148	345	196
90	22.6	4.41	73.4	19.4	202	164	308	216	248	244

**Table 5:** Capacity measured data at frequencies 113 Hz and 1 kHz.

Capacity (pF)										
	Clean insulator		L1		L2		L3		L4	
RH (%)	113 Hz	1 kHz	113 Hz	1 kHz	113 Hz	1 kHz	113 Hz	1 kHz	113 Hz	1 kHz
40	8.48	8.25	9.07	8.27	9.01	8.45	8.97	8.46	9.22	8.51
60	16.6	10.4	14.5	11	15.8	11.5	14.3	10.9	13.8	10.7
70	19	12.1	18.6	13.7	33.7	18.5	40.4	21.3	89.2	28
80	17.4	12.2	23.4	15.5	70.6	21.9	122	28.7	184	35.2
90	24.8	14.6	41.8	17.3	273	36.8	283	43.9	498	55.1

## Data Availability

The data supporting the reported results were conducted by the authors and are available on request from the corresponding author. The data are not publicly available because it is confidential.

## References

[1] Farzaneh M., Chisholm W.A. (2009). Insulators for Icing and Polluted Environments.

[2] Vosloo W.L., Macey R.E., de Tourreil C. (2004). The Practical Guide to Outdoor High Voltage Insulators.

[3] Haddad A., Warne D.F. (2004). Advances in High Voltage Engineering.

[4] Kuffel E., Zaengl W.S., Kuffel J. (2000). High Voltage Engineering: Fundamentals.

[5] Taherian R. (2019). 6-Application of Polymer-Based Composites: Polymer-Based Composite Insulators. Electr. Conduct. Polym. Based Compos..

[6] Papailiou K., Schmuck F. (2013). Composite Long Rod Insulators. Silicone Composite Insulators.

[7] Jiao J., Li L., Wu B., He C. (2017). Novel capacitive proximity sensors for assessing the aging of composite insulators. Sens. Actuators A Phys..

[8] Suen M.-S., Chen R. (2018). Capacitive Tactile Sensor with Concentric-Shape Electrodes for Three-Axial Force Measurement. Proceedings.

[9] Othman N., Piah M., Adzis Z. (2017). Contamination effects on charge distribution measurement of high voltage glass insulator string. Measurement.

[10] Qiao Z., Cheng L., Zhang S., Yang L., Guo C. Detection of composite insulators inner defects based on flash thermography. Proceedings of the 2017 1st International Conference on Electrical Materials and Power Equipment (ICEMPE).

[11] Li M., Jing Y., Zhang L., Li X., Huang G., Wang Z. Insulator Defect Detection Based on Ultraviolet Imaging and Acoustic Emission Signal. Proceedings of the 2020 IEEE 3rd Student Conference on Electrical Machines and Systems (SCEMS).

[12] Lasalvia L.A.B., Florentine M.T.B., Ferreira T.V., Germano A.D., da Costa E.G. Intelligent acoustic detection of defective porcelain station post insulators. Proceedings of the 2015 IEEE Electrical Insulation Conference (EIC).

[13] Liu L., Mei H., Guo C., Zhao C., Wang L. Nondestructive Testing of Porcelain Post Insulators Using Active Infrared Thermography. Proceedings of the 2017 IEEE 7th Annual International Conference on CYBER Technology in Automation, Control, and Intelligent Systems (CYBER).

[14] Ibrahim M.E., Selim F., Abd-Elhady A.M. (2022). Partial discharge performance improvement of covered conductor (CC)/high voltage insulator based electrical distribution systems. Electr. Power Syst. Res..

[15] Liu L., Mei H., Wang L., Zhao C., Guan Z. Pulsed infrared thermography to inspect the internal defects of composite insulators. Proceedings of the 2017 IEEE Electrical Insulation Conference (EIC).

[16] Zhang B., Cui Y., Zhang W., Liu S., Zheng Q., Yang S., Gao S. Voltage and electric field distribution along porcelain long rod insulator string in AC 500kV transmission line. Proceedings of the 2016 IEEE International Conference on High Voltage Engineering and Application (ICHVE).

[17] Mateus C., Barata F.A., Luis R. Effects of Broken Skirts and Pollution on Voltage Distribution for Cap and Pin Glass Insulators. Proceedings of the 2020 IEEE 14th International Conference on Compatibility, Power Electronics and Power Engineering (CPE-POWERENG).

[18] Ramesh M., Cui L., Gorur R.S. (2018). Impact of superficial and internal defects on electric field of composite insulators. Int. J. Electr. Power Energy Syst..

[19] Ramesh M., Gorur R. (2021). Stretched grid finite difference method for computation of electric field in composite insulators with defects. Electr. Power Syst. Res..

[20] Stefenon S.F., Corso M.P., Nied A., Perez F.L., Yow K., Gonzalez G.V., Leithardt V.R.Q. (2021). Classification of insulators using neural network based on computer vision. IET Gener. Transm. Distrib..

[21] Stefenon S.F., Ribeiro M.H.D.M., Nied A., Mariani V.C., Coelho L.D.S., Leithardt V.R.Q., Silva L.A., Seman L.O. (2021). Hybrid Wavelet Stacking Ensemble Model for Insulators Contamination Forecasting. IEEE Access.

[22] Stefenon S.F., Seman L.O., Neto N.F.S., Meyer L.H., Nied A., Yow K.-C. (2022). Echo state network applied for classification of medium voltage insulators. Int. J. Electr. Power Energy Syst..

[23] Othman N.A., Piah M.A.M., Adzis Z. Leakage current and trapped charge characteristics for glass insulator string under contaminated conditions. Proceedings of the 2015 IEEE Conference on Energy Conversion (CENCON).

[24] Deb S., Ghosh R., Dutta S., Dalai S., Chatterjee B. Effect of humidity on leakage current of a contaminated 11 kV Porcelain Pin Insulator. Proceedings of the 2017 6th International Conference on Computer Applications in Electrical Engineering-Recent Advances (CERA).

[25] Fauziah D., Alfiadi H., Rachmawati, Suwamo The effect of coating on leakage current characteristic of coast field aged ceramic insulator. Proceedings of the 2017 4th International Conference on Electrical Engineering, Computer Science and Informatics (EECSI).

[26] De Santos H., Bobi M.A.S. (2020). A Cumulative Pollution Index for the Estimation of the Leakage Current on Insulator Strings. IEEE Trans. Power Deliv..

[27] Samakosh J.D., Mirzaie M. (2019). Analysis of leakage current characteristics during aging process of SiR insulator under uniform and longitudinal non-uniform pollution conditions. Measurement.

[28] Kim T., Yi J. (2022). Application of hydrophobic coating to reduce leakage current through surface energy control of high voltage insulator. Appl. Surf. Sci..

[29] Mohammadnabi S., Rahmani K. (2021). Influence of humidity and contamination on the leakage current of 230-kV composite insulator. Electr. Power Syst. Res..

[30] Castillo-Sierra R., Oviedo-Trespalacios O., Candelo-Becerra J.E., Soto J.D., Calle M. (2021). A novel method for prediction of washing cycles of electrical insulators in high pollution environments. Int. J. Electr. Power Energy Syst..

[31] Putra N.R.M., Sartika N., Rachmawati, Suwarno The study on leakage current waveform characteristics and computer simulation of ceramic insulator under artificial tropical condition. Proceedings of the 2018 12th International Conference on the Properties and Applications of Dielectric Materials (ICPADM).

[32] Rasamsetti S.S., Sumathi N. Leakage current characteristics of 132kV polymeric and porcelain insulator under various polluted conditions. Proceedings of the 2017 IEEE International Conference on Power, Control, Signals and Instrumentation Engineering (ICPCSI).

[33] Kordkheili H.H., Abravesh H., Tabasi M., Dakhem M., Abravesh M.M. (2010). Determining the probability of flashover occurrence in composite insulators by using leakage current harmonic components. IEEE Trans. Dielectr. Electr. Insul..

[34] Ahmad H., Salam M., Ying L.Y., Bashir N. (2008). Harmonic components of leakage current as a diagnostic tool to study the aging of insulators. J. Electrost..

[35] Salem A.A., Abd-Rahman R., Al-Gailani S.A., Salam Z., Kamarudin M.S., Zainuddin H., Yousof M.F.M. (2020). Risk Assessment of Polluted Glass Insulator Using Leakage Current Index Under Different Operating Conditions. IEEE Access.

[36] Palangar M.F., Mirzaie M. (2016). Detection of Critical Conditions in Ceramic Insulators Based on Harmonic Analysis of Leakage Current. Electr. Power Compon. Syst..

[37] Salem A.A., Abd-Rahman R., Al-Gailani S.A., Kamarudin M.S., Ahmad H., Salam Z. (2020). The Leakage Current Components as a Diagnostic Tool to Estimate Contamination Level on High Voltage Insulators. IEEE Access.

[38] Bashir N., Ahmad H., Suddin M.S. Ageing studies on transmission line glass insulators using dielectric dissipation factor test. Proceedings of the 2010 Conference Proceedings IPEC.

[39] Gerdinand F., Budde M., Kurrat M. Electrical and mechanical strength of mineral filled epoxy insulators in correlation to power loss factor. Proceedings of the 2004 IEEE International Conference on Solid Dielectrics, 2004. ICSD 2004.

[40] Hu R., Zhao A., Yu C., Shi Y., Zhang X. Measurement of dielectric loss factor based on LabVIEW signal processing module. Proceedings of the 2020 2nd International Conference on Machine Learning, Big Data and Business Intelligence (MLBDBI).

[41] Levitskaya T.M., Sternberg B.K. (2019). Parameters describing the material behavior in an electromagnetic field. Electrical Spectroscopy of Earth Materials.

[42] Chrzan K., Pohl Z., Kowalak T. (1989). Hygroscopic properties of pollutants on HV insulators. IEEE Trans. Electr. Insul..

[43] Chrzan K. (1987). Conductivuty of Aqueous Dust Solutions. IEEE Trans. Electr. Insul..

